# Excision of the Whole Lower Jaw

**Published:** 1844-04-20

**Authors:** 

**Affiliations:** Padua


*Excision of the whole Lower Jaw.* By Dr. SIGNORONI, of Padua. The case of excision of the whole lower jaw, related by Dr. Bartolomeo Signoroni, is interesting as showing that patients may recover after removal of the whole lower jaw, and preserve the power of swallowing and the faculty of speech. In this case, on account of an osteo-sarcomatous affection, the whole jaw was removed at its articulations. The patient speedily recovered, and was a few months afterwards exhibited at Padua, to the Italian Scientific Association. He had completely gained his health, swallowed easily, and his speech was scarcely defective. It is much to be regretted that the details of this case are not given; it may, however, be gathered from the remarks which follow the simple announcement of its success, that the bone was extracted piece-meal, being divided into portions by means of the cutting pliers introduced through subcutaneous incisions. It is to this mode of operating the author attributes the small quantity of blood lost, the rapidity of the healing process, and the general success of the operation.—*Edin. Med. and Sur. Jour.*, *from Annali Universali di Medicini*, Feb. 1843.

